# Long-Term Immunogenicity Study of an Aluminum Phosphate-Adjuvanted Inactivated Enterovirus A71 Vaccine in Children: An Extension to a Phase 2 Study

**DOI:** 10.3390/vaccines12090985

**Published:** 2024-08-29

**Authors:** Nan-Chang Chiu, Chien-Yu Lin, Charles Chen, Hao-Yuan Cheng, Erh-Fang Hsieh, Luke Tzu-Chi Liu, Cheng-Hsun Chiu, Li-Min Huang

**Affiliations:** 1 Department of Pediatrics, MacKay Children’s Hospital, Taipei 10449, Taiwan; ncc88@mmh.org.tw; 2 Department of Medicine, Mackay Medical College, New Taipei City 25245, Taiwan; 3 Department of Pediatrics, Hsinchu Municipal MacKay Children’s Hospital, Hsinchu City 300, Taiwan; 4 Medigen Vaccine Biologics Corp., Taipei 11493, Taiwan; 5 College of Science and Technology, Temple University, Philadelphia, PA 19140, USA; 6 Department of Pediatrics, Chang Gung Memorial Hospital, Chang Gung University College of Medicine, Taoyuan City 33305, Taiwan; 7 Department of Pediatrics, National Taiwan University Children’s Hospital, Taipei City 100226, Taiwan

**Keywords:** enterovirus, enterovirus vaccine, EV-A71, EV-A71 vaccine, hand, foot and mouth disease, pediatrics, clinical trial, immune durability

## Abstract

Enterovirus A71 (EV-A71) causes hand, foot, and mouth disease in infants and children with potential for fatal complications such as encephalitis and acute flaccid myelitis. This study examined the long-term immunity conferred by EV71vac, an inactivated EV-A71 vaccine adjuvanted with aluminum phosphate, in children from the age of 2 months to <6 years, for up to 5 years after the first immunization. A total of 227 participants between 2 months and <6 years of age who had previously received either EV71vac or placebo in the phase two clinical study were enrolled. Subjects were divided into age groups: 2 years to <6 years (Group 2b), 6 months to <2 years (Group 2c), and 2 months to <6 months (Group 2d). At Year 5, the neutralizing antibody titers against the B4 subgenotype remained high at 621.38 to 978.20, 841.40 to 1159.93, and 477.71 to 745.07 for Groups 2b, 2c, and 2d, respectively. Cross-neutralizing titers at Year 5 remained high against B5 and C4a subgenotypes, respectively. No long-term safety issues were reported. Our study provides novel insights into the long-term immunity conferred by EV71vac in children aged from two months to six years, particularly in those who received EV71vac between two and six months of age.

## 1. Introduction

Enterovirus is a diverse group of single-stranded, positive-sense RNA viruses that include important human pathogens such as rhinovirus, poliovirus, coxsackievirus A, and enterovirus A71 [1,2]. Enterovirus A71 (EV-A71) is a highly contagious and significant member of the enterovirus family, causing periodic outbreaks of hand, foot, and mouth disease (HFMD) in infants and children, particularly in tropical Asian regions [1,3]. In severe cases, it can lead to serious or fatal complications such as acute flaccid myelitis, aseptic meningitis, and encephalitis [4]. The case fatality rate for patients diagnosed with EV-A71-associated HFMD in the Asia–Pacific region ranges from less than 0.5% to 19% [4].

The epidemiology of EV-A71 in the Asia–Pacific region follows a cyclic pattern, with major outbreaks occurring in cycles of 3 to 4 years, especially during the summer [4]. Currently, there are no US Food and Drug Administration (FDA) or European Medicines Agency (EMA)-approved vaccines available for EV-A71. The first three licensed inactivated whole-virus vaccines were developed and approved for use only in China based on the C4a subgenotype by the Chinese Academy of Medical Sciences (CAMS), Sinovac Biotech, and Beijing Vigoo [5]. The World Health Organization (WHO) has subsequently developed recommendations to ensure the quality, safety, and efficacy of inactivated EV-A71 vaccines, based on the previously approved vaccines [6].

EV71vac is a formalin-inactivated, aluminum phosphate-adjuvanted EV-A71 vaccine based on the B4 subgenotype. It is the first EV-A71 vaccine for use in children as young as two months of age and has been shown to provide cross-reactivity against B5, C4a, and C5 subgenotypes [7]. In a large phase three clinical trial involving approximately 3000 participants from multiple regions, the vaccine demonstrated 100% efficacy (96.8% in the Poisson regression model) in its target population [8]. The vaccine has been approved for use in children aged two months to six years in Taiwan in April 2023 and is currently under regulatory process in Vietnam [9,10]. The current study is an extension follow-up study to our phase two clinical study to investigate the long-term immunity conferred by EV71vac in children aged from two months to less than six years. Participants from the phase two trial were monitored for neutralizing antibody titers against EV-A71 for up to five years after the first vaccination by the following age groups: 2 to <6 years, 6 months to <2 years, and 2 to <6 months.

## 2. Materials and Methods

### 2.1. Study Design and Participants

This study was an extension study to the phase two main study of the EV71vac, which was a double-blind, randomized, placebo-controlled study to evaluate the safety and immunogenicity of children aged two months to 11 years (www.clinicaltrials.gov NCT 02200237) [7]. In the main study, the subjects were grouped by age into four groups, of which three groups were followed up on during this extension study: Group 2b (2 years to <6 years), Group 2c (6 months to <2 years), and Group 2d (2 months to <6 months). Subjects had previously received either EV71vac at 1.25 μg (LD), 2.5 μg (MD), 5 μg (HD) or placebo (phosphate buffer saline with adjuvant aluminum phosphate at 150 μg/0.5 mL) and did not receive any further treatment in this extension study. Group 2a from the phase two main study was excluded from the extension study as the age group (aged 6 years to <12 years) is not the target population of the vaccine but was used in the main study to confirm safety for age de-escalation. Participants in Groups 2b and 2c received two doses at 28 days apart (Days 1 and 28), while Group 2d received two doses at 56 days apart (Days 1 and 57). Groups 2c and 2d received a booster dose one year after the first dose (Day 366). Inclusion criteria were subjects who completed participation in the original phase two study in Groups 2b, 2c, and 2d, and have received protocol-specified doses of EV71vac or placebo (total of 2 doses in Group 2b, and 3 doses for Groups 2c and 2d), and that the subjects’ guardians were able to understand and sign informed consent.

The extension study was conducted from August 2019 to December 2021. The first extension study visit was 4 years ± 60 days after the first vaccination for Group 2b, and 3 years ± 60 days after the first dose for Groups 2c and 2d. Subjects of Group 2b remained in the study for approximately 12 months and had 2 clinic visits; subjects of Groups 2c and 2d remained in the study for approximately 24 months and had 3 clinic visits. Immunogenicity against EV-A71 virus antigen at each visit was assessed. The clinical trial is registered at ClinicalTrials.gov with the identifier: NCT04072276

### 2.2. Randomization and Blinding

The current extension study has no further randomization because the randomization and blinding process was performed in the phase two main study to determine the allocation of dosage groups [7]. 

### 2.3. Procedure and Outcomes

The primary objective was to evaluate the long-term antibody titers of EV7vac at 4 years and 5 years after the first dose vaccination for subjects at the age of 2 years to <6 years (Group 2b), and 3 years to 5 years after the first dose vaccination for subjects at the age of 2 months to <2 years (Groups 2c and 2d). The primary endpoints were to evaluate the immunogenicity and serum-neutralizing antibody titers (B4 subgenotype) in terms of:

- Geometric mean titer (GMT) of EV-A71 neutralizing antibody titers at 4 years and 5 years after the first dose of vaccination for part 2b; and 3 years to 5 years after the first dose of vaccination for parts 2c and 2d.
- Seroprotection rate (defined as neutralizing antibody titer ≥1:32) at 4 years and 5 years after the first dose of vaccination for part 2b; and 3 years to 5 years after the first dose of vaccination for parts 2c and 2d.

The secondary objective was to evaluate the potential of cross-protection of the EV71 vaccine. Thus, the secondary endpoints were the neutralizing antibody titers against the non-vaccine subgenotypes (B5 and C4a) through GMT of neutralizing antibody titers against the above subgenotypes. Neutralizing antibody levels against EV-A71 subgenotypes B4, B5, and C4a were assayed using a cytopathogenic effect assay (CPE) as previously described [7].

### 2.4. Statistical Analysis

The population sets used in the study for statistical analysis were the following:

Intent-to-follow (ITF) population:

- The ITF population included all enrolled subjects for whom data are available.

According-To-protocol (ATP) population:

- The ATP population for analysis of immunogenicity included all evaluable subjects (i.e., those meeting all eligibility criteria and complied with the procedures defined in the protocol during the study) for whom data concerning immunogenicity endpoint measures are available.

Safety evaluation was performed on the ITF population. Analysis of efficacy endpoints was performed on ATP. The main conclusion was made on the ATP population analysis results of the primary endpoints. The sample size was derived from the participants of the phase two clinical trial who were invited to remain in the extension study and was not derived from the statistical estimating method.

All results are presented using descriptive statistics. Neutralizing antibody titers below the cut-off value were given the value of half the cut-off for calculation purposes. All results were presented using descriptive statistics. Kruskal–Wallis test with corrected Dunn’s multiple comparisons test was used to compare the means between groups (2-tailed, alpha = 0.05). Seroprotection rate (SPR) was defined as neutralizing antibody titer ≥1:32. The 95% confidence interval values were calculated with a two-sided unpaired t-test for GMT and binomial distribution estimation for SPR. The analysis was performed using Prism 6.01 (GraphPad Software, Boston, MA, USA). 

## 3. Results

**Figure 1:**
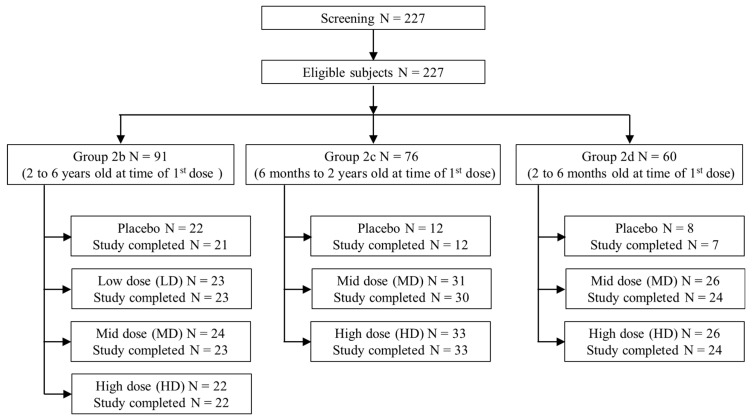
CONSORT flow diagram for the study.

The study period for the extension study was from 27 May 2016, when the first participant attended the first visit, to 11 December 2021, when the last participant finished the last visit. A total of 227 subjects were screened in this study; no subject was excluded via screening, resulting in 227 enrolled subjects, including 91 subjects in Part 2b, 76 subjects in Part 2c, and 60 subjects in Part 2d (Figure 1). All 227 eligible subjects belonged to the ITF population and ATP population. All except eight subjects (two in Part 2b, one in Part 2c, and five in Part 2d) completed the study. The demographic characteristics of the study population are summarized in Table 1. 

**Figure 2:**
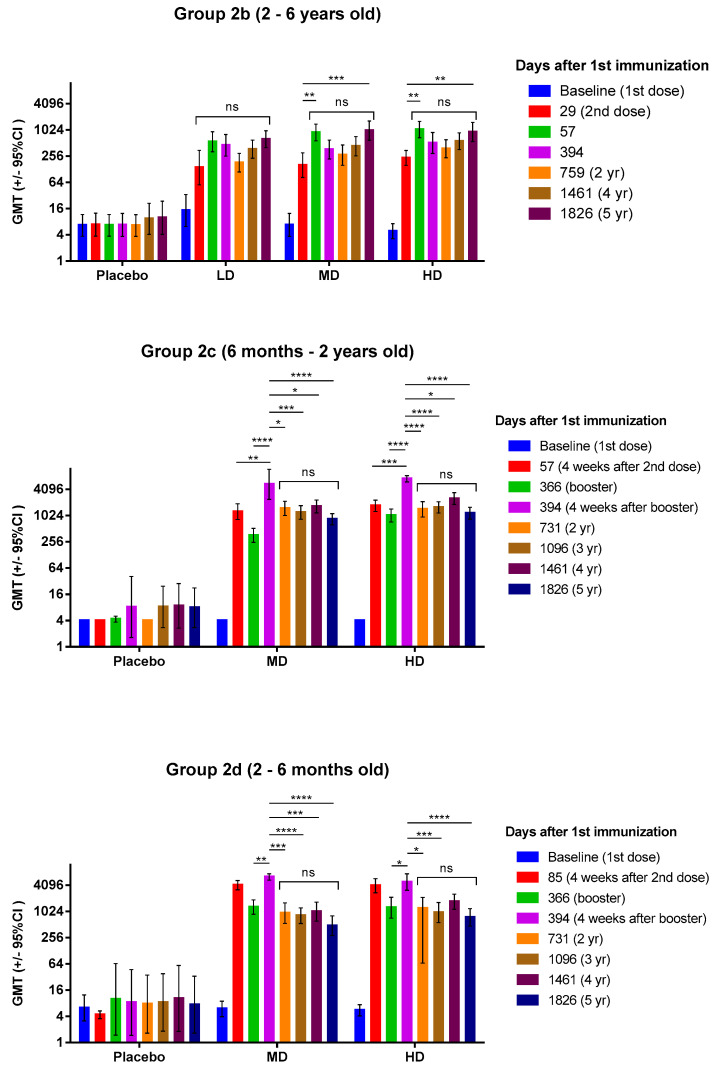
Immunogenicity of EV71vac. Anti-B4 subgenotype neutralizing antibody titers for Groups 2b, 2c, and 2d at various time points from the start of the main study (baseline) to five years after the first vaccination. The dose levels used were LD (1.25 μg), MD (2.5 μg), and HD (5 μg), and only Group 2b received all three dose levels, whereas Groups 2c and 2d only received MD and HD. Kruskal–Wallis test with corrected Dunn’s multiple comparisons test was used to compare the means between groups * = *p* < 0.05, ** = *p* < 0.01, *** = *p* < 0.001, **** = *p* < 0.0001. An ns above the bracket indicates that the GMT values contained within the bracket were statistically not significant to each other.

For Group 2b, at Year 4, the GMTs (95% CI) of EV71 B4 subgenotype-specific virus neutralizing antibody (NAb) were 367.30 (95% CI 227.23~593.70), 430.38 (258.53~716.49), and 558.24 (359.16~867.66) in subjects vaccinated with LD, MD, and HD, respectively (Figure 2 and Table 2). One year later at Year 5, the GMTs of the vaccinated subjects remained high at 621.38 (398.89~967.96), 978.20 (588.34~1626.40), and 910.48 (549.55~1508.47) for LD, MD, and HD groups, respectively. For Group 2c, at Year 3, the GMTs were 1206.99 (842.55~1729.05) and 1576.81 (1171.27~2122.77) for MD and HD, respectively, and further increased at Year 4 to 1653.18 (1178.10~2319.83) and 2501.62 (1837.26~3406.21) for MD and HD, respectively (Figure 2 and Table 2). However, at Year 5, the GMT values dropped to 841.40 (624.89~1132.92) and 1159.93 (849.04~1584.64) for MD and HD, respectively. A similar trend was observed in Group 2d, where the GMTs increased from 813.72 (538.80~1228.92) to 1007.61 (609.16~1666.71) for MD from Year 3 to Year 4, and from 956.17 (564.73~1618.93) to 1699.14 (1141.13~2530.02) for HD (Figure 2 and Table 2). At Year 5 the titers decreased to 477.71 (284.84~801.17) and 745.07 (469.41~1182.60) for MD and HD, respectively. 

The dynamic of NAb levels for all groups at each visit throughout the study is shown in Figure 2. Compared to the earlier stages of vaccination during the phase two main study, no significant increase or decrease was noted from 394 days after the first dose to the end of the study period (Figure 2 and Table S1). In all groups, NAb titers peaked at four weeks after the final dose (2nd dose for Group 2b, and booster dose for Groups 2c and 2d), and subsequently decreased but remained high until the fifth year (Figure 2). NAb titers for individual participants that had serum samples taken at all visits were plotted as line plots to show the time course of the NAb titers (Figure S1). Almost all individuals experienced some form of increased NAb titers at one point after the last immunization (Figure S1).

In terms of seroprotection rate (SPR) against the B4 subgenotype, Group 2b and 2c’s subjects remained completely seroprotected (100%) at Year 5 in all dose levels (Table 3). In Group 2d, one subject in the MD dose group had dropped to become not-seroprotected, thus resulting in an SPR of 95.8%, but the SPR of the HD group remained at 100% (Table 3). Interestingly, SPR in placebo groups increased during the course of the study to 14.3–19.0% at Year 5 (Table 3).

**Figure 3:**
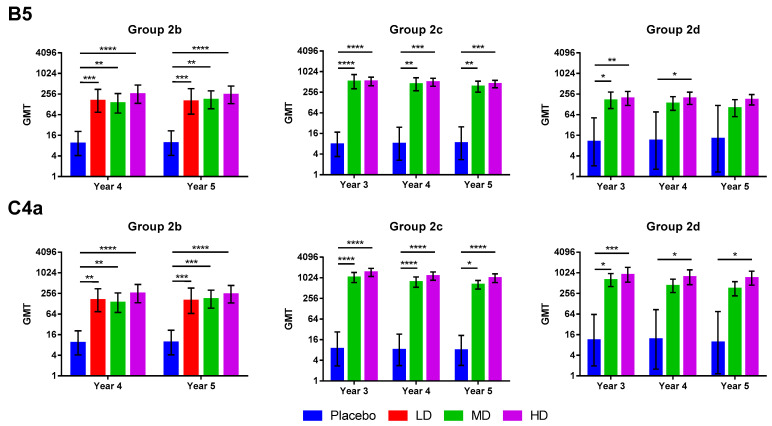
Cross-neutralizing immunogenicity of EV71vac. Neutralizing antibody titers for B5 and C4a subgenotypes were shown for Groups 2b, 2c, and 2d at three to five years after the first vaccination. The dose levels used were LD (1.25 μg), MD (2.5 μg), and HD (5 μg), and only Group 2b received all three dose levels, whereas Groups 2c and 2d only received MD and HD. Results are expressed as bars representing GMT and error bars representing a 95% confidence interval. Kruskal–Wallis test with corrected Dunn’s multiple comparisons test was used to compare the means between groups * = *p* < 0.05, ** = *p* < 0.01, *** = *p* < 0.001, **** = *p* < 0.0001.

NAb titers against B5 and C4a subgenotypes also remained high throughout the study period. At Year 5, the GMTs against B5 ranged from 156.77 to 242.78, 372.59 to 444.30, and 99.14 to 174.63 for Groups 2b, 2c, and 2d, respectively (Figure 3 and Table S2). At Year 5, the GMTs against C4a ranged from 341.94 to 436.72, 641.68 to 998.20, and 350.60 to 717.39 for Groups 2b, 2c, and 2d, respectively (Figure 3 and Table S3). 

For long-term safety evaluation, physical examinations were performed at all visits. Overall, relatively few subjects presented medical conditions in both placebo and vaccine groups. As these medical conditions occurred years after vaccination, these were deemed less likely to be related to the vaccine. No particular trends were observed between Placebo and each EV71 vaccine dose group (Table S4).

## 4. Discussion

In this study, we investigated the long-term immunity for up to five years after the first dose of EV71vac in children aged from two months to six years. To our knowledge, this is the first study of long-term immunity imparted by the EV-A71 vaccine involving children aged from two to six months, which makes the findings significant in filling this knowledge gap.

Our data have shown that the antibody elicited by EV71vac persisted throughout the five years of the study period against not only the B4 vaccine subgenotype but also other subgenotypes. Clinical evidence from our phase three efficacy trial also suggested that EV71vac cross-protected against non-B4 subgenotypes [8]. The vaccine can therefore provide a strong foundation for protection against EV-A71 through infancy and childhood. The finding is crucial, considering that the age group of two to six months is particularly vulnerable to severe complications from EV-A71 infection. In the previous phase three study, the number of subjects who reported adverse events was similar in the EV71vac and placebo group during the 14-month observation period, and almost all reported solicited adverse events were mild and self-limited [8]. The results of this extension study further suggest that this EV71 vaccine is safe up to 5 years after the first dose as investigated by clinical visits. 

Our results are in line with the current knowledge regarding the durability of post-vaccination antibody levels, which show that antibody titers peaked at one month after the last vaccination, and remained stable at much higher levels than pre-vaccination [8]. It has been found that long-term antibody maintenance after exposure or vaccination is mainly attributed to the following: (1) short-lived plasma cells are triggered by re-exposure to antigens which causes memory B cells to divide and differentiate; and (2) long-lived plasma cells, which are derived from germinal center B cells and reside in bone marrow and secret high-affinity antibodies without re-exposure to antigens (LLPCs) [10,11]. 

We have observed that the GMTs against the B4 subgenotype of Groups 2c and 2d at all dosage levels increased from Year 3 to Year 4, but decreased at Year 5 (Figure 2 and Table S1). The changes in GMTs from Year 3 to Year 5 were not statistically significant; however, the same trend was not observed in GMTs against B5 and C4a subgenotypes, where the GMTs show a decreasing trend from Year 3 to Year 5 (Figure 3). In contrast, the GMTs in Group 2b continuously increased from Year 2 to Year 5 instead of decreasing as seen in Groups 2c and 2d (Figure 2 and Table S1). Individual NAb titers of the participants showed that almost all vaccinees experienced increases in NAb titers after an initial decay of titer from the last dose of vaccine, while some placebo participants also had increased NAb titers during the course of study (Figure S1). As previously mentioned, this could be attributed to natural booster effect triggered by memory recall of B-cells when vaccine-primed individuals were re-exposed to the same virus in the environment [10]. In contrast to vaccine-primed participants, only a few placebo participants had increased NAb titers due to natural boosting; as placebo participants had not been primed by vaccination to the viral antigen, they did not possess the same immunological memory as vaccinees against exposure to the virus. Taiwan had higher-than-usual cases of EV-A71 in 2019, which coincided with the time between Year 3 and Year 4 in this study, and the participants may have been exposed to an increased amount of EV-A71 in the environment which resulted in an increase in NAb titers at Year 4 [12]. Some studies have found that EV-A71 can generate weak cross-reactive antibodies against coxsackievirus A (CVA) serotypes and some cross-reactive epitopes have been identified in EV-A71 against other picornaviruses [13,14]. However, studies using human sera of children infected with various enteroviruses in Vietnam have found that there is little or no cross-neutralization among EV-A71, CVA6, CVA10, and CVA16, occurring in only a handful of patients [15]. The authors of the previous study noted that the seropositive and seroconversion rates could be attributed to previous exposure or co-infection with other enteroviruses [15]. In our case, this could be one plausible explanation, as CVAs remain the predominant type of enterovirus that circulates in Taiwan, and it is possible that exposure to these CVAs can induce cross-reactive antibodies [12].

In spite of the natural boosting we have observed in this study, four participants from Groups 2c and 2d did not exhibit natural boosting as their titers continuously decreased after peak titer on Day 394 (Figure S2). At five years after the first dose, the NAb titers had an approximate 11- to 41-fold decrease compared to peak titer level (Day 394). Despite the drop in titers, they remained well above the seroprotection level, ranging from 200 to 708. These results then showed that even in the absence of natural boosting, immunization with EV71vac can provide persisting antibody immunity for at least five years after the first immunization. However, the sample size was too small to enable us to establish a robust NAb titer decay model for EV71vac.

The increase of SPR in our placebo groups was 14.3% to 19.0% in the fifth year, noticeably lower than that observed in a five-year study in China, where SPR of the placebo group increased from 4.76% to 71.43% within 5 years [16]. One Chinese study has observed dramatic waning antibody titers just 6 months after the primary series of 2 vaccinations, from 509.0–1383.2 on Day 56 to 58.8–177.4 at 8 months post-vaccination in all age and treatment groups [17]. In a subsequent study for the same vaccine as the booster, the same subjects showed that the NAb titers became stabilized and slightly increased to 138.2–264.3 at the time of the start of the booster study (one year after the first dose), and the authors also attributed the slight increase of antibody titers post-vaccination to natural booster due to re-exposure to EV-A71 [18]. Another study that investigated the immunity of the EV-A71 vaccine over a period of 2 years observed a similar natural booster phenomenon to our study, namely that participants had increased NAb titers over one year after the last vaccination, regardless of whether they were given the vaccine or placebo [19]. One potential reason why increases in SPR in placebo groups were much higher in China than in Taiwan could be that EV-A71 persistently circulates in China, while in Taiwan it causes more sporadic outbreaks [12,20].

The observed long-term immunity has important implications for public health and disease prevention. Children within the two to six months age range are at a critical stage of development and are most vulnerable to EV-A71 infection, and providing them with adequate protection against infectious diseases is paramount. By demonstrating the vaccine’s ability to induce long-lasting immunity in this age group, we contribute valuable information to guide immunization strategies and policies for infants and young children. Large-scale EV-A71 outbreaks which occur in waves are attributed to a younger naïve population and thus, vaccination of this younger population is key to preventing and breaking the cycle of EV-A71 outbreaks [21]. As of now, the EV-A71 vaccine is not part of the national immunization program in Taiwan and is being paid for out-of-pocket. To promote awareness against enterovirus infection and vaccination, health agencies and doctors in Taiwan have been encouraging parents to vaccinate their children, especially before the start of the school year [22,23,24].

We acknowledge the limitations of this study. The sample size was relatively small due to the selection of participants from the phase two trial. However, even with a relatively small sample size, the ranges of 95% confidence intervals of neutralizing antibody GMTs and SPR remain sufficiently small up to Year 5, demonstrating the homogeneity of neutralizing antibody response induced by EV71vac (Figure 2 and Figure 3). Long-term follow-up studies of efficacy in vaccinated individuals with larger cohorts could be conducted to validate and further investigate the real-world effectiveness of EV71vac against infection. The lack of immunogenicity data in the literature for the two- to six-month age group also limited our ability to compare our findings in this age group directly with previous studies. Further analysis is required to study the modeling of immune persistence, such as with half-life and the decay of neutralization antibody response. Although this study did not investigate the magnitude and durability of cellular immunity, data from prior human clinical studies with inactivated EV-A71 vaccines have shown that neutralizing antibodies induced by vaccines are strongly correlated with protection against EV-A71-associated diseases [25]. The evidence from these studies have led to the proposal of neutralizing antibody titer as the de facto standard for correlates of protection [21,25]. However, it is known that cellular immunity plays an important role in controlling EV-A71 infection and preventing severe disease outcomes, and some studies have shown that inactivated vaccines can induce robust cellular response, including B-cell and T-cell activation, and IFN-gamma-specific T-cell response with minimal activation of proinflammatory cytokines [21]. As there were few studies of EV-A71 vaccine cellular immunity, it would be of interest to examine the long-term impact and durability of cellular immunity conferred by EV71vac in future studies. 

## 5. Conclusions

As EV-A71 has a cyclical outbreak pattern and the majority of severe diseases occur in the age group of below three years, a booster dose administered after one year of the primary series in the age group of 2 months to 2 years can be used to ensure continuous protection against outbreaks, as shown in this study.

## Figures and Tables

**Table 1:** Summary of demographic characteristics of the participants at the time of screening * (ITF population).

	Group 2b (2–6 Years Old at Time of 1st Dose)				Group 2c (6 Months–2 Years Old at Time of 1st Dose)			Group 2d (2–6 Months Old at Time of 1st Dose)		
	Placebo	LD	MD	HD	Placebo	MD	HD	Placebo	MD	HD
N	22	23	24	22	12	31	33	8	26	26
Age at time of screening (Mean years, SD) *	8.5 (1.26)	9.0 (1.14)	8.2 (1.1)	8.6 (1.1)	5.5 (0.26)	5.2 (0.36)	5.2 (0.40)	4.0 (0.16)	4.1 (0.14)	4.1 (0.14)
Male/Female (number of participants)	11/11	14/9	16/8	10/12	6/6	15/16	20/13	4/4	12/14	9/17
Duration from first vaccination (Mean years, SD)	4.3 (0.07)	4.4 (0.07)	4.4 (0.07)	4.2 (0.07)	4.0 (0.09)	3.9 (0.12)	3.9 (0.12)	3.7 (0.13)	3.8 (0.14)	3.8 (0.12)

* 4 years ± 60 days after the first vaccination for Group 2b, and 3 years ± 60 days after first dose for Groups 2c and 2d.

**Table 2:** Neutralizing antibody titers against B4 subgenotype (ATP population).

	Group 2b (2–6 Years Old at Time of 1st Dose)				Group 2c (6 Months–2 Years Old at Time of 1st Dose)			Group 2d (2–6 Months Old at Time of 1st Dose)		
	Placebo	LD	MD	HD	Placebo	MD	HD	Placebo	MD	HD
Year 3 (Day 1096)										
N	-	-	-	-	12	31	33	8	26	26
GMT (95% CI) *	-	-	-	-	8.22 (2.75–24.56)	1206.99 (842.55–1729.05)	1576.81 (1171.27–2122.77)	7.64 (1.65–35.28)	813.72 (538.80–1228.92)	956.17 (564.73–1618.93)
Year 4 (Day 1461)										
N	22	23	24	22	12	31	32	7	24	25
GMT (95% CI) *	9.25 (4.06–21.08)	367.30 (227.23–593.70)	430.38 (258.53–716.49)	558.24 (359.16–867.66)	8.69 (2.68–28.17)	1653.18 (1178.10–2319.83)	2501.62 (1837.26–3406.21)	10.28 (1.80–58.92)	1007.61 (609.16–1666.71)	1699.14 (1141.13–2530.02)
Year 5 (Day 1826)										
N	21	23	23	22	12	30	33	7	24	24
GMT (95% CI) *	9.82 (4.09–23.57)	621.38 (398.89–967.96)	978.20 (588.34–1626.40)	910.48 (549.55–1508.47)	7.90 (2.78–22.43)	841.40 (624.89–1132.92)	1159.93 (849.04–1584.64)	7.40 (1.64–33.23)	477.71 (284.84–801.17)	745.07 (469.41–1182.60)

* Geometric mean titer, 95% confidence interval calculated by two-sample *t* test.

**Table 3:** Seroprotection * rate against B4 subgenotype (ATP population).

	Group 2b (2–6 Years Old at Time of 1st Dose)				Group 2c (6 Months–2 Years Old at Time of 1st Dose)			Group 2d (2–6 Months Old at Time of 1st Dose)		
	Placebo	LD	MD	HD	Placebo	MD	HD	Placebo	MD	HD
Year 3 (Day 759)										
N	-	-	-	-	12	31	33	8	26	26
Number of seroprotected participants (NAb ≥ 1:32)	-	-	-	-	2	31	33	1	26	25
SPR (95% CI **)	-	-	-	-	16.7 (2.09–48.41)	100.00 (88.78–100.00)	100.00 (89.42–100.00)	12.5 (0.32–52.65)	100.00 (86.77–100.00)	96.2 (80.36–99.90)
Year 4 (Day 1461)										
N	22	23	24	22	12	31	32	7	24	25
Number of seroprotected participants (NAb ≥ 1:32)	4	22	24	22	2	31	32	1	24	25
SPR (95% CI **)	18.18 (5.19–40.28)	95.65 (78.05–99.89)	100.00 (85.75–100.00)	100.00 (84.56–100.00)	16.7 (2.09–48.41)	100.00 (88.78–100.00)	100.00 (89.11–100.00)	14.3 (0.36–57.87)	100.00 (85.75–100.00)	100.00 (86.28–100.00)
Year 5 (Day 1826)										
N	21	23	23	22	12	30	33	7	24	24
Number of seroprotected participants (NAb ≥ 1:32)	4	23	23	22	2	30	33	1	23	24
SPR* (95% CI **)	19.05 (5.45–41.91)	100.00 (85.18–100.00)	100.00 (85.18–100.00)	100.00 (84.56–100.00)	16.7 (2.09–48.41)	100.00 (88.43–100.00)	100.00 (89.42–100.00)	14.3 (0.36–57.87)	95.8 (78.88–99.89)	100.00 (85.75–100.00)

* Seroprotective rate; seroprotective defined as neutralizing antibody titer ≥1:32. ** 95% confidence interval calculated by binomial distribution estimation.

## Data Availability

Data produced in the study and study protocols are available upon reasonable request to the author. The study protocol synopses have been included in the Supplementary Materials: CT-EV-21e (for up to two years after immunization) and FU-EV-21e (for up to five years after immunization).

## Supplementary Materials

The following supporting information containing protocol synopses, Figures S1 and S2, and Tables S1—S4 can be downloaded at: https://www.mdpi.com/article/10.3390/vaccines12090985/s1.
