# Supplementary material for: Long-Term Immunogenicity Study of an Aluminum Phosphate-Adjuvanted Inactivated Enterovirus A71 Vaccine in Children: An Extension to a Phase 2 Study
Source: Vaccines (Basel). 2024 Aug 29;12(9):985. doi: 10.3390/vaccines12090985 (PMC11435984; doi:10.3390/vaccines12090985)
Supplement: Supplementary file 1 [file vaccines-12-00985-s001.zip › vaccines-3151795 - Protocol synopsis.pdf]

PRTOCOL SYNOPSIS

**NAME OF SPONSOR**<br>Medigen Vaccine Biologics Corporation	**PROTOCOL NUMBER**:<br>CT-EV-21e
**NAME OF FINISHED PRODUCT:**<br>EV-71 vaccine	
**NAME OF ACTIVE INGREDIENT:**<br>Inactivated EV71 whole virion (EV71vac bulk)	

**Study Title**	Long-Term Safety and Immunogenicity Study of Inactivated EV71 Vaccine Produced in Vero Cells with Adjuvant $AlPO_4$ in Children: Extension Study of Protocol CT-EV-21
**Number of Centers**	2 – 5 centers
**Clinical Phase**	Phase II, Extension
**Study Products, Dose and Mode of Administration**	
**Name, Formulation and Dosing**	No vaccine administration will happen during this extension study CT-EV-21e<br>All enrolled subjects in this study have received Investigational Vaccine EV71 or Placebo Vaccine during CT-EV-21 study (total of 2 doses in part 2b, and 3 doses for part 2c or 2d)
**Study Objectives**	
**Primary Objective**	1. To evaluate the safety of the EV71vaccine; up to 2 years after 2nd vaccination for part 2b, and 1 year after booster dose of vaccination for part 2c and 2d.<br>2. To evaluate the long-term antibody titers of EV71 vaccine; up to 2 years after 2nd vaccination in part 2b, and 1 year after booster dose in part 2c and 2d.
**Secondary Objectives**	1. To evaluate the clinical efficacy of EV71vaccine in children
**Methodology**	
**Overall Study Design**	This is an extension study of part 2b, 2c, and 2d of protocol CT-EV-21, to evaluate the long-term safety, immunogenicity and clinical efficacy of EV71vaccine in children.<br><br>The first study visit will be approximately 12 months after the administration of first vaccination of EV71vac/placebo in CT-EV-21 study; 365±30 days after the 2nd vaccination for part 2b, and 365±30 days after 1st vaccination for part 2c and 2d.

	All subjects will remain in the study for approximately 1 year and will have 7 study visits including the 2 clinic visits and 5 telephone contact visits. Safety evaluation including adverse events (AE) and serious adverse events (SAE) will be recorded, immunogenicity response against EV71 virus antigen at V1 and V7 will be assessed and safety lab assay will be conducted
**Study Population and Selection Criteria**	
**Number of Subjects**	Approximately 270 subjects from the clinical trial CT-EV-21 part 2b, 2c or 2d, will be enrolled by invitation
**Study Population**	Healthy children who had received EV71/Placebo vaccination in CT-EV-21 study
**Inclusion criteria**	1. Subjects who have completed participation in study CT-EV-21 part 2b, 2c or 2d, and have received protocol specified doses of EV71 or Placebo Vaccine (total of 2 doses in part 2b, and 3 doses for part 2c or 2d).<br>2. The subjects' guardians are able to understand and sign the informed consent form.
**Exclusion Criteria**	1. Subjects and/or guardians who refuse to comply with the study procedures
**Visit Schedule**	No subject may undergo any investigation under this protocol including screening investigation, until written, signed and dated informed consent has been obtained.<br><br>The duration of study is approximately 12 months. The visit schedule will be as followed:<br><br>• Baseline visit (Day 1, V1): for inclusion/exclusion criteria confirmation and safety procedures. Vital signs will be checked. Medical history will be reviewed. Blood sample (5 ml) will be obtained for lab and immunogenicity assay for part 2b subjects only. Baseline lab and immunogenicity assay for part 2c and 2d will adopt results assessed at V7 (the booster dose) from CT-EV-21 study.<br>• Month 2 telephone visit (Day 60, V2): Telephone contact to evaluate the safety and events of enterovirus infection.<br>• Month 4 telephone visit (Day 120, V3): Telephone contact to evaluate the safety and events of enterovirus infection.<br>• Month 6 visit (Day 180, V4): Telephone contact to evaluate the safety and events of enterovirus infection.

	• Month 8 telephone visit (Day 240, V5): Telephone contact to evaluate the safety and events of enterovirus infection.<br>• Month 10 telephone visit (Day 300, V6): Telephone contact to evaluate the safety and events of enterovirus infection.<br>• Month 12 visit (Day 365, V7): blood sample (5 mL) will be obtained for lab and immunogenicity assay.<br>• HFMD confirmation visit: For collection of throat, and/or rectal swabs/stool culture, per the clinical signs (if symptoms are consistent with HFMD or enterovirus infection throughout 12-month period during the study).
**Study Endpoints**	
**Primary Endpoint**	1. Overall safety of EV71 vaccine in children throughout the study period, regarding:<br>• Occurrence, intensity and relationship to vaccination of adverse events and serious adverse events during the entire period of study<br>• Vital signs and Laboratory tests including complete blood cell count/differentiated count (CBC/DC), Blood Urea Nitrogen (BUN), Creatinine, and Alanine Aminotransferase (ALT).<br><br>2. Long term immunogenicity; serum neutralizing antibody titers, humoral immune response, induced by EV71 vaccine after 1~2 years, in terms of:<br>• Geometric mean titer (GMT) of EV71 neutralizing antibody titers; 1 year and 2 years after 2nd vaccination for part 2b; and 1 year after booster dose for part 2c and 2d.<br>• Seropositivity rate: (defined as Neutralizing Antibody titer ≥1:8); 1 year and 2 years after 2nd vaccination for part 2b; and 1 year after booster dose for part 2c and 2d<br>• Seroprotection rate (SPR, defined as neutralizing antibody titer ≥1:16): at 1 year and 2 years after 2nd vaccination for part 2b; and 1 year after booster dose for part 2c and 2d.
**Secondary Endpoints**	1. Clinical efficacy of EV71 vaccine by measuring:<br>• The occurrence of EV71-associated hand, foot, and mouth disease or, herpangina, or other EV71-associated diseases.<br>• The hospitalization rate of EV-71-associated HFMD, herpangina, or other EV71-associated diseases.
**Statistical Considerations**	
**General Statistical Methodology**	The focus is to evaluate the safety and immunogenicity of the candidate vaccine. All results will be presented using descriptive statistics. Significance tests (2-tailed, alpha=0.05) will be performed where appropriate and p-values will be rounded to three decimal places if applied.

	Regarding descriptive statistics, continuous variables will be summarized with number of subject, mean, standard deviation, median, range as well as 95% confidence interval, IQR, and 95% confidence interval based on median; while number and percentage of subject for categorical variables.
**Sample Size**	Up to 270 subjects

NAME OF SPONSOR<br><br>Medigen Vaccine Biologics Corporation	PROTOCOL NUMBER:<br><br>FU-EV-21e
**Study Title**	Extension study of Protocol CT-EV-21 to examine the long-term immunogenicity after receiving inactivated EV-71 vaccine produced in Vero Cells with Adjuvant AlPO4 in Children
**Number of Centers**	2-4 centers
**Clinical Phase**	Follow-up Phase II Extension Study
**Study Products, Dose and Mode of Administration**	
**Name, Formulation and Dosing**	No vaccine administration will be given to subjects during this study.
**Study Objectives**	
**Primary Objective**	To evaluate the long-term antibody titers of EV71 vaccine 4 and 5 years after first dose vaccination for subjects at the age of 2 to < 6 years , and 3 to 5 years after first dose vaccination for subjects at the age of 2 months to < 2 years
**Secondary Objectives**	To evaluate the potential of cross-protection of EV71 vaccine.
**Methodology**	
**Overall Study Design**	This is an extension study of part 2b, 2c, and 2d of protocol CT-EV-21, to evaluate the long-term immunogenicity of EV71vaccine.<br><br>The first study visit will be approximately 3 years/4 years after the administration of first vaccination of EV71 vaccine or placebo in CT-EV-21 study; 4 years±60 days after the first vaccination for subjects 2 to < 6 years of age (part 2b), and 3 years±60 days after first dose for subjects 2 months to < 2 years of age (part 2c and 2d).<br><br>Subjects of part 2b will remain in the study for approximately 12 months and will have 2 clinic visits; subjects of part 2c and 2d will remain in the study for approximately 24 months and will have 3 clinic visits. Immunogenicity response against EV71 virus antigen at each visit will be assessed.

Study Population and Selection Criteria

**Number of Subjects**	Approximately 320 subjects from the clinical trial CT-EV-21 part 2b, 2c, and 2d will be enrolled by invitation
**Study Population**	Healthy children who had received EV71/Placebo vaccination in CT-EV-21 study
**Inclusion Criteria**	1. Subjects who have completed participation in study CT-EV-21 part 2b, 2c, and 2d, and have received protocol specified doses of EV71 vaccine or Placebo (total of 2 doses in part 2b, and 3 doses for part 2c and 2d).<br>2. The subjects' guardians are able to understand and sign the informed consent form.
**Exclusion Criteria**	1. Subjects and/or guardians who refuse to comply with the study procedures
**Visit Schedule**	No subject may undergo any investigation under this protocol, including screening investigation, until written, signed and dated informed consent has been obtained.<br>For part 2b, the duration of study is approximately 12 months.<br>For part 2c and 2d, the duration of study is approximately 24 months.<br>The visit schedule will be as followed:<br>• Baseline visit (Day 1, V1):<br>Inclusion/exclusion criteria confirmation. Physical examination. Specific medication will be reviewed. Blood sample (5 ml) will be obtained for immunogenicity and cross-protection assay.<br>• Month 12 visit (Day 365, V2):<br>Physical examination. Specific medication will be reviewed. Blood sample (5 mL) will be obtained for immunogenicity and cross-protection assay.<br>• Month 24 visit (Day 730, V3):<br>Physical examination. Specific medication will be reviewed. Blood sample (5 mL) will be obtained for immunogenicity and cross-protection assay (For part 2c and 2d only).

Study Endpoints

**Primary Endpoint**	To evaluate immunogenicity, serum neutralizing antibody titers (genotype: B4) humoral immune response, induced by the investigational vaccine, in terms of:

	• Geometric mean titer (GMT) of EV71 neutralizing antibody titers at 4 and 5 years after first dose of EV71 vaccination for part 2b; and 3 to 5years after first dose of EV71 vaccination for part 2c and 2d.<br>• Seroprotection rate (defined as Neutralizing Antibody titer ≥1:32) at 4 and 5 years after first dose of EV71vaccination for part 2b; and 3 to 5years after first dose of EV71 vaccination for part 2c and 2d.
**Secondary Endpoint**	To evaluate the potential of cross-protection of EV-71 vaccine: by measuring the neutralizing antibody titers of other subgenotypes of EV-71 strains, performed on each visit

Statistical Considerations

**General Statistical Methodology**	The focus is to evaluate the long-term immunogenicity of EV71 vaccine. All results will be presented using descriptive statistics. Significance tests (2-tailed, alpha=0.05) will be performed where appropriate and p-values will be rounded to three decimal places if applied.<br>Regarding descriptive statistics, continuous variables will be summarized with number of subject, mean, standard deviation, median, range as well as 95% confidence interval, IQR, and 95% confidence interval based on median; while number and percentage of subject for categorical variables.
**Sample Size**	Up to 320 subjects
