# Supplementary material for: Long-Term Immunogenicity Study of an Aluminum Phosphate-Adjuvanted Inactivated Enterovirus A71 Vaccine in Children: An Extension to a Phase 2 Study
Source: Vaccines (Basel). 2024 Aug 29;12(9):985. doi: 10.3390/vaccines12090985 (PMC11435984; doi:10.3390/vaccines12090985)
Supplement: Supplementary file 1 [file vaccines-12-00985-s001.zip › vaccines-3151795 - Supplements v3.pdf]

Figure S1. Individual neutralizing antibody titers in participants who had serum samples taken in all visits.

Individual neutralizing antibody titers from all participants that had serum samples taken in all visits were plotted from the first immunization (baseline) to 5 years after the first immunization. A single dot represents the NAb titer of an individual at a given time point and is connected with lines to all time points.

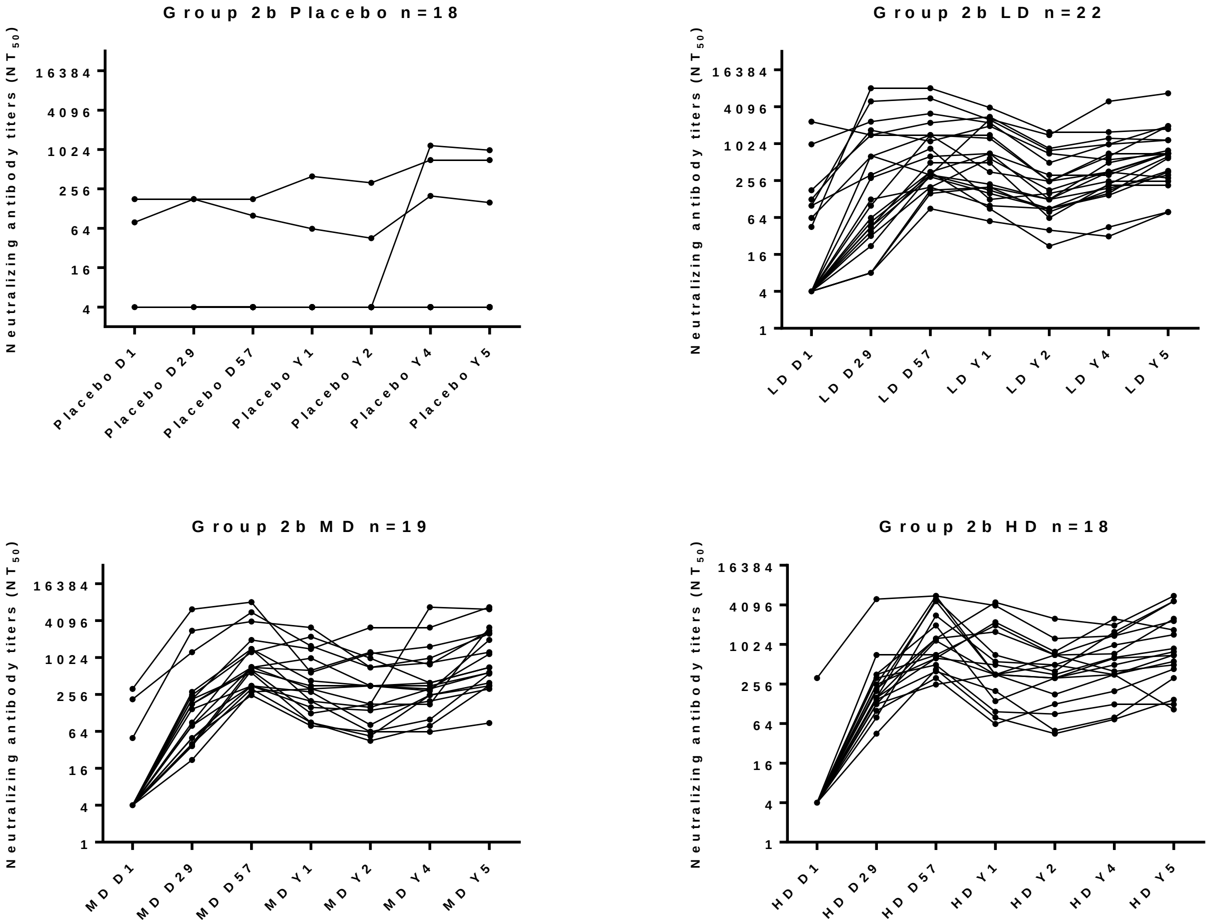

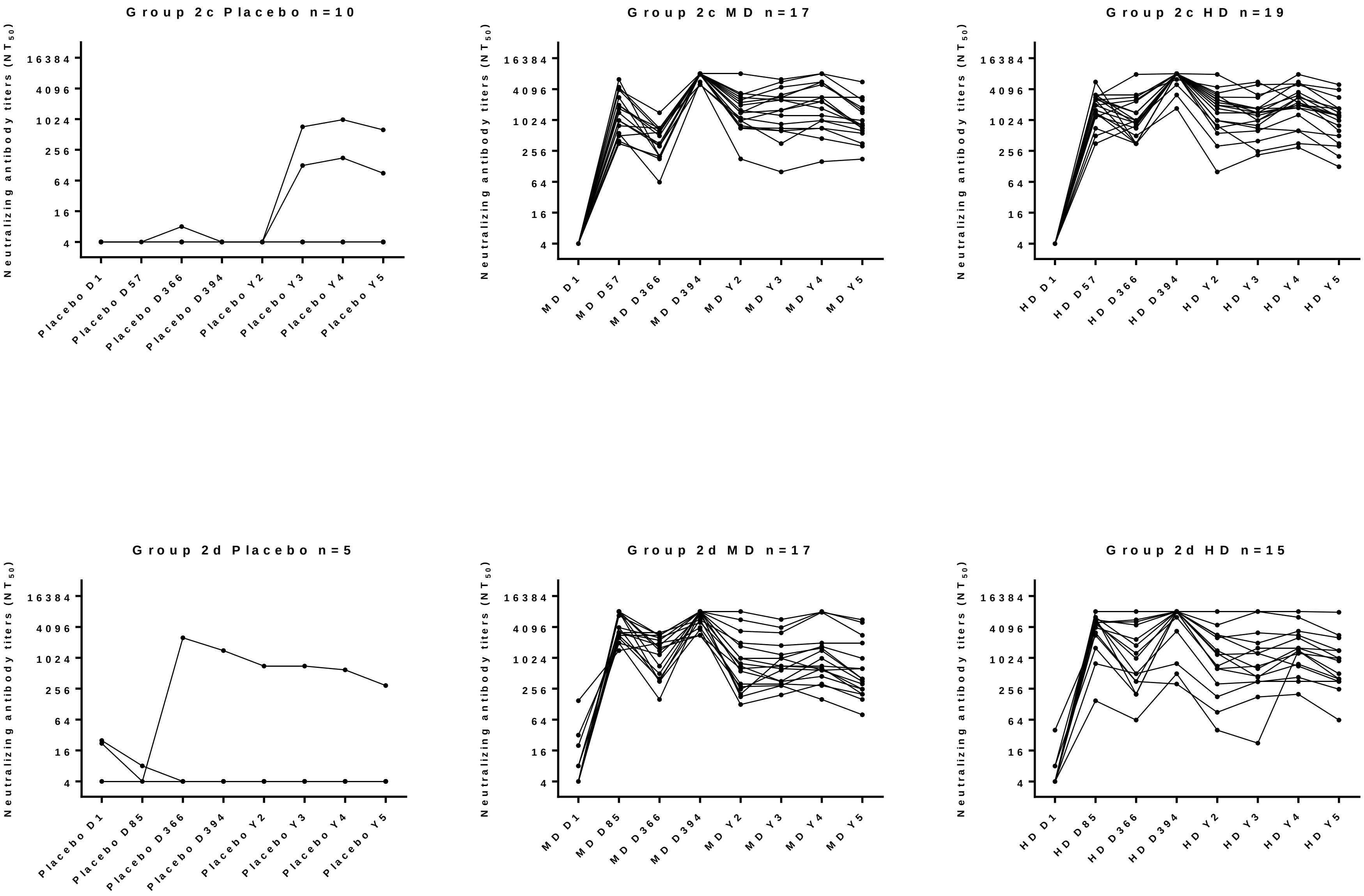

Group 2c Placebo n=10
Group 2c MD n=17
Group 2c HD n=19
Group 2d Placebo n=5
Group 2d MD n=17
Group 2d HD n=15
Neutralizing antibody titers (NT50)
16384
4096
1024
256
64
16
4
Placebo D1
Placebo D57
Placebo D85
Placebo D366
Placebo D394
Placebo Y2
Placebo Y3
Placebo Y4
Placebo Y5
MD D1
MD D57
MD D85
MD D366
MD D394
MD Y2
MD Y3
MD Y4
MD Y5
HD D1
HD D57
HD D85
HD D366
HD D394
HD Y2
HD Y3
HD Y4
HD Y5

Figure S2. Neutralizing antibody titers in individuals with continuous decreasing titers after peak titer at Day 394.

Individual neutralizing antibody titers from four participants in Groups 2c and 2d (n=2 from 2c MD, n=1 from 2c HD, and n=1 from 2d MD) were plotted from the first immunization (baseline) to 5 years after the first immunization.

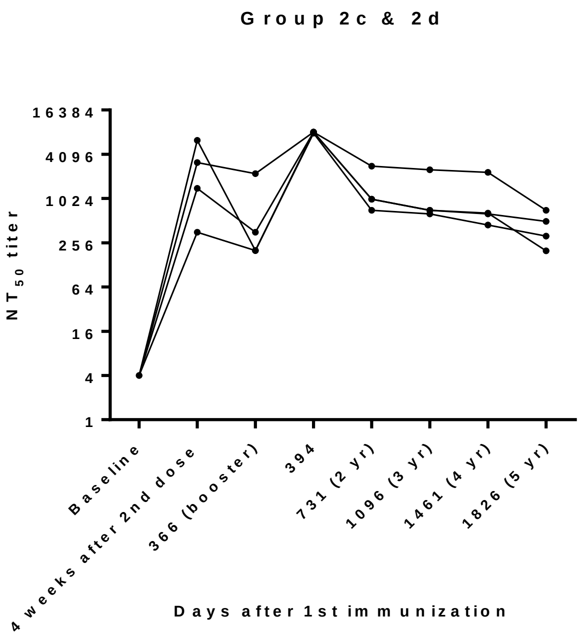

Table S1. Neutralizing antibody titers and seroprotection rate against B4 subgenotype from early stages of vaccination (ATP population)

	**Group 2b**				**Group 2c**			**Group 2d**		
	**Placebo**	**LD**	**MD**	**HD**	**Placebo**	**MD**	**HD**	**Placebo**	**MD**	**HD**
**Baseline**	-									
N	23	24	21	23	10	20	23	8	22	24
GMT (95% CI*)	**6.56** (3.69 –	**14.41** (6.19 –	**6.71** (3.66 –	**4.84** (3.26 -	**4.00** (4.00 –	**4.00** (4.00 –	**4.00** (4.00 –	**6.22** (3.14 –	**5.94** (3.96 –	**5.49** (4.09 –

	11.65)	33.55)	12.33)	7.17)	4.00)	4.00)	4.00)	12.35)	8.91)	7.37)
# of SPR (NAb ≥ 1: 32)	3	8	3	1	0	0	0	2	2	2
Seroprotection rate (95% CI**)	**13.04** (2.78 – 33.59)	**33.33** (15.63 – 55.32)	**14.29** (3.05 – 36.34)	**4.35** (0.11 – 2.19)	**0.00** (0.00 – 30.85)	**0.00** (0.00 – 16.84)	**0.00** (0.00 – 14.82)	**25.00** (3.19 – 65.09)	**9.09** (1.12 – 29.16)	**8.33** (1.03 – 27.00)
	**Prior to 2nd dose (Day 29)**				**28 days after 2nd dose (Day 57)**			**28 days after 2nd dose (Day 85)**		
N	23	24	21	23	10	20	23	8	22	24
GMT (95% CI*)	**6.8** (3.7 – 12.5)	**138.59** (55.55 – 345.76)	**157.45** (82.22 – 301.51)	**231.54** (156.75 – 342.02)	**4.00** (4.00 – 4.00)	**1256.08** (830.57 – 1899.61)	**1713.27** (1267.82 – 2315.24)	**4.36** (3.55 – 5.35)	**4075.30** (3177.56 – 5226.66)	**3957.93** (2728.31 – 5741.72)
# of SPR (NAb ≥ 1: 32)	3	18	20	23	0	20	23	0	22	24
Seroprotection rate (95% CI**)	**13.04** (2.78 – 33.59)	**72.00** (50.61 – 87.93)	**95.24** (76.18 – 99.88)	**100.00** (85.18 – 100.00)	**0.00** (0.00 – 30.85)	**100.00** (83.16 – 100.00)	**100.00** (85.18 – 100.00)	**0.00** (0.00 – 36.94)	**100.00** (84.56 – 100.00)	**100.00** (85.75 – 100.00)
	**28 days after 2nd dose (Day 57)**				**Prior to booster dose (Day 366)**			**Prior to booster dose (Day 366)**		
N	23	24	21	23	10	20	23	8	22	24
GMT (95% CI*)	**6.56** (3.71 – 11.60)	**544.43** (320.86 – 923.78)	**885.16** (568.02 – 1379.38)	**1033.99** (668.37 – 1599.63)	**4.29** (3.67 – 5.02)	**358.57** (247.82 – 518.82)	**1026.85** (727.45 – 1449.48)	**9.76** (1.48 – 64.36)	**1281.18** (875.59 – 1874.66)	**1239.88** (713.02 – 2156.05)
# of SPR (NAb ≥ 1: 32)	3	24	21	23	0	20	23	1	22	24
Seroprotection rate (95% CI**)	**13.04** (2.78 – 33.59)	**100.00** (85.76 – 100.00)	**100.00** (83.89 – 100.00)	**100.00** (85.18 – 100.00)	**0.00** (0.00 – 30.85)	**100.00** (83.16 – 100.00)	**100.00** (85.18 – 100.00)	**12.50** (0.32 – 52.65)	**100.00** (84.56 – 100.00)	**100.00** (85.75 – 100.00)
	**1 year after 2nd dose (Day 394)**				**28 days after booster dose (Day 394)**			**28 days after booster dose (Day 394)**		
N	23	24	21	23	10	20	23	8	22	24
GMT (95% CI*)	**6.71** (3.66 – 12.29)	**449.44** (254.33 – 794.26)	**360.60** (219.82 – 591.54)	**508.63** (291.42 – 887.73)	**8.16** (1.63 - 40.98)	**5333.01** (2408.10 – 11810.55)	**7106.92** (6026.83 – 8380.58)	**8.33** (1.47 – 47.19)	**6270.33** (5308.34 – 7406.65)	**4788.66** (3101.50 – 7393.59)
# of SPR (NAb ≥ 1: 32)	3	24	21	23	1	19	23	1	22	24

Seroprotection rate (95% CI**)	**13.04** (2.78 – 33.59)	**100.00** (85.76 – 100.00)	**100.00** (83.89 – 100.00)	**100.00** (85.18 – 100.00)	**10.00** (0.25 – 44.50)	**95.00** (75.13 – 99.87)	**100.00** (85.18 – 100.00)	**12.50** (0.32 – 52.65)	**100.00** (84.56 – 100.00)	**100.00** (85.75 – 100.00)
	**2 years after 2nd dose (Day 759)**				**1 year after booster dose (Day 731)**			**1 year after booster dose (Day 731)**		
N	23	24	21	23	10	20	23	8	22	24
GMT (95% CI*)	**6.51** (3.66 – 11.56)	**180.02** (110.23 – 293.99)	**267.49** (156.61 – 456.89)	**375.68** (232.85 – 606.13)	**4.0** (4.0 – 4.0)	**1490.24** (1023.84 – 2169.08)	**1421.22** (943.92 – 2139.86)	**7.64** (1.65 – 35.29)	**930.67** (540.98 – 1601.09)	**1196.47** (668.74 – 2140.64)
# of SPR (NAb ≥ 1: 32)	3	23	21	23	0	20	23	1	22	24
Seroprotection rate (95% CI**)	**13.04** (2.78 – 33.59)	**95.83** (78.88 – 99.89)	**100.00** (83.89 – 100.00)	**100.00** (85.18 – 100.00)	**0.00** (0.00 – 30.85)	**100.00** (83.16 – 100.00)	**100.00** (85.18 – 100.00)	**12.50** (0.32 – 52.65)	**100.00** (84.56 – 100.00)	**100.00** (85.75 – 100.00)

*Two-sample t test

**Binomial distribution estimation

Table S2. Neutralizing antibody titers against B5 subgenotype (ATP population).

	**Group 2b (2 – 6 years old at time of 1st dose)**				**Group 2c (6 months – 2 years old at time of 1st dose)**			**Group 2d (2 – 6 months old at time of 1st dose)**		
	**Placebo**	**LD**	**MD**	**HD**	**Placebo**	**MD**	**HD**	**Placebo**	**MD**	**HD**
**Year 3 (Day 759)**										
N	-	-	-	-	12	31	33	8	26	26
GMT (95% CI)*	-	-	-	-	**7.72** (3.40 – 17.51)	**520.84** (323.70 – 838.04)	**528.70** (395.11 – 707.44)	**10.33** (2.06 – 51.86)	**168.44** (96.61 – 293.69)	**190.33** (118.71 – 305.15)
**Year 4 (Day 1461)**										
N	22	23	24	22	12	31	32	7	24	25
GMT (95% CI)*	**9.23** (4.11 – 20.76)	**162.61** (75.57 – 349.91)	**137.48** (71.40 – 264.70)	**253.57** (136.64 – 470.56)	**8.05** (2.66 – 24.41)	**438.25** (284.00 – 676.28)	**500.45** (381.76 – 656.05)	**11.26** (1.64 – 77.07)	**135.77** (86.14 – 213.98)	**192.58** (127.73 – 290.35)
**Year 5 (Day 1826)**										
N	21	23	23	22	12	30	33	7	24	24
GMT (95% CI*)	**9.46** (4.12 – 21.70)	**156.77** (66.53 – 369.40)	**174.44** (95.38 – 319.05)	**242.78** (134.57 – 437.98)	**8.29** (2.75 – 24.96)	**372.59** (257.36 – 539.42)	**444.30** (345.73 – 570.96)	**12.68** (1.35 – 118.92)	**99.14** (56.14 – 175.05)	**174.63** (123.16 – 247.62)

*Geometric mean titer, 95% confidence interval calculated by two-sample t test.

Table S3. Neutralizing antibody titers against C4a subgenotype (ATP population).

	Group 2b (2 – 6 years old at time of 1st dose)				Group 2c (6 months – 2 years old at time of 1st dose)			Group 2d (2 – 6 months old at time of 1st dose)		
	**Placebo**	**LD**	**MD**	**HD**	**Placebo**	**MD**	**HD**	**Placebo**	**MD**	**HD**
**Year 3 (Day 759)**			-							
N	-	-	-	-	12	31	33	8	26	26
GMT (95% CI)*	-	-	-	-	**8.58** (2.73 – 26.93)	**1042.52** (744.02 – 1460.78)	**1470.06** (1117.95 – 1933.08)	**11.02** (1.95 – 62.26)	**629.22** (406.03 – 975.09)	**897.20** (545.70 – 1475.12)
**Year 4 (Day 1461)**										
N	22	23	24	22	12	31	32	7	24	25
GMT (95% CI)*	**9.66** (4.12 – 22.64)	**317.04** (190.82 – 526.75)	**313.83** (205.42 – 479.47)	**575.40** (386.64 – 856.30)	**8.05** (2.80 – 23.19)	**769.43** (544.02 – 1088.23)	**1147.36** (871.77 – 1510.06)	**11.64** (1.57 – 86.00)	**426.00** (271.14 – 669.32)	**760.19** (461.95 – 1250.97)
**Year 5 (Day 1826)**										
N	21	23	23	22	12	30	33	7	24	24
GMT (95% CI)*	**9.56** (4.12 – 22.16)	**341.94** (208.89 – 559.74)	**353.69** (222.40 – 562.47)	**436.72** (277.37 – 687.61)	**7.79** (2.84 – 21.37)	**641.68** (480.27 – 857.32)	**998.20** (742.61 – 1341.75)	**9.40** (1.16 – 76.09)	**350.60** (216.88 – 566.76)	**717.39** (447.75 – 1149.41)

*Geometric mean titer, 95% confidence interval calculated by two-sample t test.

Table S4. Safety physical examination (ITF population)

	**Group 2b**				**Group 2c**			**Group 2d**		
	**Placebo**	**LD**	**MD**	**HD**	**Placebo**	**MD**	**HD**	**Placebo**	**MD**	**HD**
**Year 3 (Day 1096)**										
**N**	-	-	-	-	**12**	**31**	**33**	**8**	**26**	**26**
**At least one of below**	-	-	-	-	**2**	**1**	**5**	**2**	**0**	**0**
Chest					1	0	0	0	0	0
Eyes, ENT, Mouth & Tongue					2	0	3	1	0	0
Respiratory					2	1	1	1	0	0
Skin, Nails & Hair					0	0	1	1	0	0
**Year 4 (Day 1461)**										
**N**	**22**	**23**	**24**	**22**	**12**	**31**	**32**	**7**	**24**	**25**
**At least one of below**	**1**	**0**	**0**	**0**	**0**	**2**	**1**	**0**	**0**	**0**
Chest	0	0	0	0	0	1	0	0	0	0

Eyes, ENT, Mouth & Tongue	1	0	0	0	0	1	0	0	0	0
Respiratory	0	0	0	0	0	0	1	0	0	0
Skin, Nails & Hair	0	0	0	0	0	0	0	0	0	0
**Year 5 (Day 1826)**										
**N**	**21**	**23**	**23**	**22**	**12**	**30**	**33**	**7**	**24**	**24**
**At least one of below**	**1**	**0**	**1**	**1**	**0**	**0**	**0**	**0**	**0**	**0**
Chest	1	0	0	0	0	0	0	0	0	0
Eyes, ENT, Mouth & Tongue	0	0	1	0	0	0	0	0	0	0
Respiratory	0	0	0	0	0	0	0	0	0	0
Skin, Nails & Hair	0	0	0	1	0	0	0	0	0	0
